# Analysis of Internet Usage Among Cancer Patients in a County Hospital Setting: A Quality Improvement Initiative

**DOI:** 10.2196/resprot.2806

**Published:** 2014-05-09

**Authors:** Lucy Wallace, Lisa Lilley, William Lodrigues, Julie Dreadin-Pulliam, Xian-Jin Xie, Sakshi Mathur, Madhu Rao, Valorie Harvey, Ann Marilyn Leitch, Roshni Rao

**Affiliations:** ^1^University of Texas SouthwesternDallas, TXUnited States; ^2^Parkland Memorial HospitalDallas, TXUnited States

**Keywords:** Internet, cancer, quality, quality improvement, patient education

## Abstract

**Background:**

Cancer is one of the most common diseases that patients research on the Internet. The Commission on Cancer (CoC) recommended that Parkland Memorial Hospital (PMH) improve the oncology services website. PMH is Dallas County’s public health care facility, serving a largely uninsured, minority population. Most research regarding patient Internet use has been conducted in insured, Caucasian populations, raising concerns that the needs of PMH patients may not be extrapolated from available data. The PMH Cancer Committee, therefore, adopted a quality improvement initiative to understand patients’ Internet usage.

**Objective:**

The objective of the study was to obtain and analyze data regarding patients’ Internet usage in order to make targeted improvements to the oncology services section of the institutional website.

**Methods:**

A task force developed an 11-question survey to ascertain what proportion of our patients have Internet access and use the Internet to obtain medical information as well as determine the specific information sought. Between April 2011 and August 2011, 300 surveys were administered to newly diagnosed cancer patients. Multivariate analyses were performed.

**Results:**

Of 300 surveys, 291 were included. Minorities, primarily African-American and Hispanic, represented 78.0% (227/291) of patients. Only 37.1% (108/291) of patients had Internet access, most (256/291, 87.9%) having access at home. Younger patients more commonly had Internet access, with a mean age of 47 versus 58 years for those without (*P*<.001). Education beyond high school was associated with Internet access (*P*<.001). The most common reason for Internet research was to develop questions for discussion with one’s physician. Patients most frequently sought information regarding cancer treatment options, outcomes, and side effects.

**Conclusions:**

Less than one-half of PMH oncology patients have Internet access. This is influenced by age, educational level, and ethnicity. Those with access use it to obtain information related to their cancer diagnosis. The most effective way of addressing our patients’ needs using the institutional website is to provide links to reputable disease-specific sites.

## Introduction

Cancer is among the top three diseases that the public researches on the Internet [1]. Studies reveal that up to 63% of cancer patients search the Internet for information about their diagnosis [2]. Common reasons patients search the Internet are to develop questions to discuss with their physicians, verify information already received from their physicians, and seek alternative treatment options [2]. Research reveals significant differences in Internet usage based on age [2], education [3], income [3], and ethnicity [3-6].

Hospitals are facing increasing pressure to initiate and maintain an Internet presence, particularly in competitive markets where consumers have the resources and ability to compare and select their preferred health care organizations [7]. Additionally, accrediting organizations, such as the American College of Surgeons Commission on Cancer (CoC), encourage participating members to create and maintain an Internet presence that includes patient education materials as well as information about the hospital itself.

Parkland Memorial Hospital (PMH) is Dallas County’s public health care facility. It serves a largely uninsured, minority, and uneducated population, particularly with regard to cancer programs [8,9]. In March 2009, the CoC advised PMH to improve the presence of oncology services on the institutional website and include patient educational materials. A task force was subsequently formed, comprised of physicians, hospital administrators, nurses, and patient advocates, in order to oversee this quality improvement initiative.

A review of the literature was performed and revealed that the majority of research regarding patient Internet use was performed in insured, primarily Caucasian populations [2-4]. This raised concerns that the educational needs of these patients could not be extrapolated to the population that PMH serves. In addition, it was unclear what proportion of the PMH cancer patients actually had Internet access and would use it to seek medical information. Therefore, due to the paucity of data pertinent to the patterns of use and educational needs of the PMH patient population, a patient survey was developed. At the time of our study, prior surveys had been conducted, but in primarily Caucasian, insured populations. Therefore, the lack of such a survey in a minority, uninsured population prevented direct adoption of previously used instruments. It is worth noting that there were few investigations available for review that focused on the Internet use in the Hispanic population, which is the fastest growing demographic in the United States and a considerable segment of the PMH patient population. More recent studies of Internet usage in this rapidly growing segment of the population have focused primarily on mobile text messaging [5,6]. While insightful and compelling, these are not directly applicable to the CoC mandate for PMH to improve the oncology services presence and provide educational materials on the institutional website.

Prior to expending valuable resources, it was critical to understand how resources would be best applied. The goals of the survey were to ascertain rates of Internet access, rates of medical Internet use, and to understand the specific educational needs among the PMH cancer population. This information would be used to make targeted improvements to the oncology services section of the PMH institutional website.

## Methods

### Recruitment

After discussion with the institutional review board (IRB), because the primary focus of the study was quality improvement, this project was approved and initiated as an IRB-exempt protocol. From April 2011 to August 2011, 300 surveys were administered to newly diagnosed cancer patients presenting to PMH oncology clinics. Patients still without a known cancer diagnosis, those presenting for follow-up, and patients who were not yet 18-years old were excluded from the study. The surveys were administered in clinics focusing on breast, gastrointestinal, liver, lung, prostate, and gynecological cancers to provide broad representation and include the most commonly treated cancers at PMH. Survey administration was performed in the clinics by medical assistants, both in an attempt to ensure the surveys were completed as thoroughly as possible and so that illiterate and non-English speaking patients would not be inadvertently excluded. Patients who only spoke Spanish were administered the survey by Spanish speaking staff. During the months of survey administration, April through August of 2011, 864 patients were eligible to participate. These patients were approached during their initial oncology clinic visit and, of these, 300 patients agreed to participate, yielding a participation rate of 34.7% (300/864). Most of those who chose not to participate cited time constraints. No additional questioning or data collection was performed regarding reasons for nonparticipation.

### Data Collection

The survey primarily focused on whether patients had Internet access, the location of Internet usage, and whether they attempted to obtain information on the Internet about their diagnosis. Demographic data including age, gender, race, and educational level was collected. Patients were asked to provide their specific cancer diagnosis. Additionally, to obtain guidance on the content of the PMH oncology services website, information regarding the patients’ educational needs was also collected. Patients were asked if their primary reason for performing Internet research was to develop questions for discussion with their physician, verify information already received from their physician, investigate alternative treatments, or to learn about PMH. Patients were asked to select the type of information they were seeking and were given the following choices: cancer treatment options, cancer treatment outcomes, resources for coping with cancer, cancer clinical trials, cancer treatment side effects, and information about PMH. Patients were asked how many minutes per day were spent conducting Internet research. They were also asked if they would use the PMH site more if the content were offered in Spanish.

### Statistical Analysis

De-identified patient information was collected and entered in an Excel spreadsheet for analysis. Fisher exact test was used for categorical data. Multivariate analyses were performed to determine which factors significantly impacted Internet access rates.

## Results

### Patient Surveys

Of the 300 surveys that were administered throughout PMH oncology clinics, 291 were sufficiently completed for inclusion into the study. Analysis revealed that 107/291 patients (36.8%) had Internet access. The majority of these, 94/107 patients (87.9%), had Internet access at home. Of all patients with access to the Internet, 70/107 patients (65.4%), reported that they used the Internet to research their cancer diagnosis.

### Demographics

Demographic data for the overall study population are depicted in Table 1. Most of the study population study population, 200/291 patients (68.7%), had obtained some or all of a high school education. Only 20/291 patients (6.9%) had graduated from college and an even fewer, 9/291 patients (3.1%), had obtained a graduate degree. The majority, 227/291 patients (78.0%), were minorities, primarily African-American and Hispanic.

The data were organized according to Internet access. Multivariate analyses were performed and the results are depicted in Table 2. Younger patients were more likely to have Internet access. The mean age of patients with Internet access was 47 versus 58 years for those without (*P*<.001). Patients with an educational level beyond high school had Internet access more often than those with a high school diploma or less (*P*<.001). There was no difference in Internet access rates among males and females. There was no statistically significant difference in rates of Internet access among the various races represented.

**Table 1:** Study population demographic data.

Demographics		n (%)
**Gender**		
	Male	163 (56.0)
	Female	118 (40.5)
	Missing Data	110 (3.4)
**Race**		
	African-American	108 (37.1)
	Hispanic	96 (32.9)
	Caucasian	54 (18.6)
	Asian	16 (5.5)
	Other	7 (2.4)
	Missing Data	10 (3.4)
**Educational Level**		
	High School	200 (68.7)
	Beyond High School	78 (26.8)
	Some College	48 (16.8)
	Some Graduate School	20 (6.9)
	Graduate Degree	9 (3.1)
	Missing Data	13 (4.5)
**Cancer Type**		
	Genitourinary Tract	51 (17.5)
	Gynecologic	44 (15.1)
	Gastrointestinal Tract	43 (14.8)
	Breast	41 (14.1)
	Lung	28 (9.6)
	Other	56 (19.2)
	Missing Data	28 (9.6)

**Table 2:** Multivariate analyses.

Factor/variable analysis		Odds ratio	95% CI	*P* value
**Age**				
	Continuous	1.083	1.053-1.114	<.001
**Gender**				
	Female	Reference^a^		
	Male	2.491	1.043-5.951	.0399
**Race**				
	Asian, Caucasian	0.475	0.232-0.971	.0414
	African-American, Hispanic	Reference		
**Education**				
	High School	Reference		
	>High School	0.194	0.095-0.397	<.001
**Cancer Type**				
	Breast	4.577	1.488-14.081	.008
	Gastrointestinal	2.236	0.716-6.986	.1661
	Lung	5.816	1.509-22.414	.0105
	Gynecologic	Reference		
	Genitourinary	1.480	0.399-5.490	.5578
	Other	5.301	1.757-15.997	.0031

^a^The word reference is used to delineate the variable against which the others were compared to obtain the statistical data displayed.

### Internet Usage

The qualitative data regarding patients’ reasons and goals for their Internet use is represented in Table 3 and discussed herein. For the analysis of survey questions exploring the reasons for performing Internet research and the specific information that was sought, only the 107 surveys reporting positive Internet access were used. Also, multiple answers were allowed. There were 57.0% (61/107) of the patients who stated that the primary goal of their Internet research was to assist them in developing questions for discussion with their physician, this being the most common response. There were 38/107 (35.5%) patients who stated they used the Internet to verify information already received. Only 14.9% (16/107) patients stated their primary goal was to learn about PMH. The most common categories of information that patients are seeking on the Internet are information regarding cancer treatment options (42/67, 63%), cancer treatment outcomes (30/57, 53%), and cancer treatment side effects (23/49, 46%). Very few (7/28, 26%) were interested in learning about cancer trials. Even fewer (1/14, 13%) were seeking information specific to PMH.

When asked how many minutes were spent using the Internet daily, the most common response was 30 minutes. Of 107 patients, 25 (23.4%) stated that they would use the PMH oncology services website more if the information were also presented in Spanish. All of those patients were Hispanic and that number represents 61% (25/41) of the Hispanic patients who reported having Internet access.

**Table 3:** Patient goals for Internet use.

Patient goals		n (%)
**Reason for performing Internet research**		
	Develop questions for discussion with physician	61 (57.0)
	Learn about PMH	16 (14.9)
	Investigate alternative treatments	37 (34.6)
	Verify information already received	38 (35.5)
**Specific information sought**		
	Cancer treatment options	67 (62.6)
	Cancer treatment outcomes	57 (53.3)
	Information about PMH	14 (13.1)
	Coping with cancer	41 (38.3)
	Cancer trials	28 (26.2)
	Cancer treatment side effects	49 (45.8)

## Discussion

### Principal Findings

Less than one-half of PMH oncology patients have access to the Internet. This is lower than Internet access rates of 80% previously reported in study populations that are primarily Caucasian and insured [2]. However, the proportion of PMH patients with Internet access was greater than anticipated, supporting an effort to improve the oncology services presence on the PMH institutional website. This is especially true given that, of the patients with Internet access, the percentage who actually use the Internet to research their cancer diagnosis is comparable with that which has been reported in study populations that are primarily Caucasian and insured, 42% to 63% [2,3].

Regarding the portion of the survey that addressed content, a pattern emerged in the responses. Patients expressed little interest in information specific to PMH. It is believed that this likely speaks to the fact that PMH patients, being enrolled in Dallas County’s public health care system, do not have other options for health care delivery, and therefore are not using the Internet to compare hospitals.

Patients’ primary reason for performing Internet research was to develop questions for discussion with their physicians. They were most interested in finding information regarding cancer treatment options, outcomes, and side effects. This is what the task force has chosen to focus on, as changes have been made to the Oncology Services portion of the PMH institutional website. It was determined that the most efficient and cost-effective way to address the CoC recommendation, to provide educational materials on the institutional website, is to provide links to the websites of other reputable disease-specific organizations, such as American Cancer Society, National Institutes of Health, and Susan G Komen for the Cure.

### Limitations

The inherent limitation of this and any study using a survey to gather data is that there is no way to account for recall bias on the part of the respondent. There were also surveys that were used but had some missing data. In other words, despite the survey being administered by a medical assistant in person, there were patients who did not complete every single question on the survey. An additional limitation of the survey format is that only the particular responses that are included in the answer choices can be analyzed. This survey did not include a section for free-form responses and, in the occasional instance of narrative response, there was no mechanism for analysis.

### Conclusions

The findings of this quality improvement initiative will serve to assist other like institutions working to improve Internet resources for patients in an efficient and cost-effective manner. This is particularly true as it relates to other hospitals with a large and growing Hispanic population, as it has already been stated that this is the fastest growing demographic in the United States and was well represented in this study population.

In general, there is more pressure for health care systems to create and maintain a presence on the Internet, particularly in competitive markets in which patients have many choices. There is also the regulatory obligation for hospitals to implement and maintain an Internet presence, as the CoC mandate illustrates. However, this study has shown that in the county hospital setting, serving primarily minority, uninsured patients, valuable resources need not be dedicated to elaborate website interface implementation and maintenance. By conducting this survey and understanding the ways patients use the Internet and the specific information they are seeking, the PMH task force was able to successfully satisfy the CoC requirement using relatively few resources.

## Abbreviations

CoC: Commission on Cancer
IRB: institutional review board
PMH: Parkland Memorial Hospital
